# Mechanism of Mutation in G Protein-Gated Inwardly Rectifying K^+^ Channel in Familial Hyperaldosteronism-Type III: Residue Fluctuations and Conformational Instability

**DOI:** 10.3390/molecules31111842

**Published:** 2026-05-27

**Authors:** Asmaa S. AbuMaziad, Julia J. Liang, Alex N. O. Logothetis, Eleni Pitsillou, Andrew Hung, Jordan Beck, Rissa Zudekoff, Autri Hafezi, Bruce Chy, Abigail Slack, AbdAssalam Qannus, Assam El-Osta, Tom C. Karagiannis

**Affiliations:** 1 Department of Pediatrics, Division of Nephrology, University of Arizona College of Medicine, Tucson, AZ 85724, USA; 2 Thomas W. Keating Bioresearch Building, BIO5 Institute, The University of Arizona, Tucson, AZ 85721, USA; 3 EpiMed Centre, Baker Heart and Diabetes Institute, Melbourne, VIC 3004, Australia; 4 Baker Department of Cardiovascular Research Translation and Implementation, La Trobe University, Melbourne, VIC 3086, Australia; 5 Department of Microbiology and Immunology, The University of Melbourne, Parkville, VIC 3010, Australia; 6 School of Science, STEM College, RMIT University, Melbourne, VIC 3001, Australia; 7 Department of Medicine, Division of Nephrology, University of Arizona Health Sciences, Tucson, AZ 85721, USA; 8 Epigenetics in Human Health and Disease Program, Baker Heart and Diabetes Institute, Melbourne, VIC 3004, Australia; sam.el-osta@baker.edu.au; 9 Department of Diabetes, Central Clinical School, Monash University, Melbourne, VIC 3004, Australia; 10 Department of Medicine and Therapeutics, The Chinese University of Hong Kong, Sha Tin, Hong Kong SAR, China; 11 Hong Kong Institute of Diabetes and Obesity, Prince of Wales Hospital, The Chinese University of Hong Kong, Sha Tin, Hong Kong SAR, China; 12 Li Ka Shing Institute of Health Sciences, The Chinese University of Hong Kong, Sha Tin, Hong Kong SAR, China; 13 Baker Department of Cardiometabolic Health, The University of Melbourne, Parkville, VIC 3010, Australia; 14 Department of Clinical Pathology, The University of Melbourne, Parkville, VIC 3010, Australia

**Keywords:** hypertension, familial hypertension, primary aldosteronism, *KCNJ5*, G protein-activated inward-rectifier potassium channel, GIRK4

## Abstract

Primary aldosteronism (PA) is the most common cause of secondary hypertension and accounts for 5–15% of hypertensive patients. Familial hyperaldosteronism, a monogenic cause of PA, accounts for ~1–5% of cases. Familial hyperaldosteronism type III results from mutations in the *KCNJ5* gene, which lead to excessive aldosterone production and hypertension due to dysfunction of the GIRK4 channel in the adrenal gland. Despite the importance of *KCNJ5* in PA pathogenesis, little is known about the molecular mechanisms underlying germline *KCNJ5* mutations and their functional consequences. This study explored the structural changes in *KCNJ5* pathogenic variant c.452G>A (p.Gly151Glu or GIRK4^G151E^). Homology modeling and molecular dynamics simulations of the mutant GIRK4 channel showed that structural rearrangements occur in GIRK4^G151E^ when compared to GIRK4^WT^, displaying higher RMSD and SASA, which may be attributed to differences in residue fluctuations in the cytosolic and extracellular domains, and ligands may bind with a stronger affinity to GIRK4^G151E^. Given that the mutation is located within or proximal to the selectivity filter of GIRK4, we expect that the primary mechanism of dysfunction involves altered ion selectivity, leading to membrane depolarization. Our novel findings highlight the importance of understanding the molecular mechanisms underlying *KCNJ5* mutations in PA and hypertension pathogenesis. This knowledge could inform the development of more targeted and effective treatments for this condition.

## 1. Introduction

Hypertension is a major risk factor for cardiovascular disease, stroke, and death. The etiology of hypertension is complex and multifactorial, with both genetic and environmental factors contributing to its development. Primary aldosteronism (PA) is the leading cause of endocrine hypertension and affects around 5–15% of patients with secondary hypertension [1,2,3]. Overproduction of aldosterone characterizes this condition, and the most common causes of primary aldosteronism are bilateral adrenal hyperplasia, which accounts for 60–70%, aldosterone-producing adenoma, which accounts for 30–40%, and familial hyperaldosteronism, which contributes to about 5–7% of cases. All forms of primary aldosteronism are due to an underlying somatic or germline mutation.

Aldosterone, synthesized by the zona glomerulosa of the adrenal cortex, is tightly controlled to regulate blood pressure and electrolyte balance. In the kidneys, aldosterone binds to the mineralocorticoid receptor (MR) in the distal tubules, and its main role is the regulation of sodium reabsorption and potassium excretion. Aldosterone directly stimulates epithelial sodium channels in the collecting duct (ENaC), sodium–potassium ATPase, and potassium channels (ROMK) to maximally increase sodium reabsorption, which will lead to increased water uptake, thereby increasing blood pressure and altering the potassium balance in the body [4].

Patients with PA, characterized by hypertension in the setting of high aldosterone levels, are at high risk for cardiac, vascular, central nervous system, and renal complications. Left ventricular hypertrophy, myocardial infarction, heart failure, atrial fibrillation, and cerebral stroke are frequently reported in these patients [5,6]. Hyperkalemia is observed in about 30% of patients who have PA. The common symptoms related to low potassium levels include heart palpitations, excessive fatigue, and severe muscle weakness.

Four forms of familial PH, I–IV, have been described. The known genetic syndromes featuring primary aldosteronism are autosomal dominant familial hyperaldosteronism types I, II, III, and IV with variable age of onset, but mostly childhood and adolescence. FH-I, also known as glucocorticoid-remedial aldosteronism (GRA), is found in ~5% of PA patients. FA-I is caused by a chimeric gene of CYP11B1 and CYP11B2, which results from an unequal crossover event. The *CYP11B2* gene encodes the enzyme aldosterone synthase, while *CYP11B1* encodes 11 β-hydroxylase, which catalyzes the final step of cortisol synthesis [2,3,4,5,6,7]. As a result of carrying a chimeric gene with two parts of two different enzymes, ACTH stimulates both 11 β-hydroxylase and aldosterone synthase, leading to aldosterone production/secretion [6] independent of normal physiological angiotensin II regulation. FH Type II, a nonglucocorticoid-remediable form of primary aldosteronism, is inherited in an autosomal dominant fashion and sporadically. The genetic defect has been localized to chromosome 3 and is related to the chloride channel, *CLCN2* [4,6]. When mutated, this gene predisposes the glomerulosa zone cell to depolarize and increase intracellular calcium, thereby resulting in elevated CYP11β2 (aldosterone synthase) transcription and expression, which is responsible for aldosterone synthesis. FH-IV is caused by pathogenic variants in *CACNA1H*, which encodes a T-type calcium channel Cav3.2. The mutation is a gain-of-function type that causes slower inactivation and prolonged opening of the calcium channel, leading to increased influx of calcium into the cell and a greater and more prolonged stimulus for transcription of the *CYP11B2* gene responsible for aldosterone synthesis [8,9]. These patients often develop early-onset hypertension and sometimes developmental delay. Primary aldosteronism with seizures and neurologic abnormalities (PASNA) is caused by de novo germline pathogenic variants in CACNA1D and has been reported in three children, so it is exceedingly rare.

FH-III is a distinctive form of primary aldosteronism (PA) characterized by its unique biochemical properties and clinical presentation [10,11,12]. The first case of this condition was reported by Lifton’s group in 2008, which was notable for its relatively early and severe onset in childhood [13].

The defect in FH-III is caused by pathogenic variants in the potassium inwardly rectifying channel subfamily J member 5 gene or *KCNJ5* located on chromosome 11q24.3 (composed of three exons), which encodes G protein-regulated inward-rectifier potassium channel type 4 (GIRK4). GIRK4 channels are part of a superfamily of GIRK channels that includes seven family members where only four GIRK subunits, GIRK1-4 (also designated Kir3.1-4), have been identified in mammals [14,15]. GIRK1, 2, and 3 are abundant in the brain and heart, while GIRK4 is limited to the adrenal and pituitary glands. The role of GIRK channels is to maintain the resting membrane potential near the potassium equilibrium potential to regulate cellular excitability and to maintain the K homeostasis that leads to hyperpolarization and a reduction in membrane excitability.

It is not yet clear how *KCNJ5* mutations work at the molecular level. The molecular mechanisms of *KCNJ5* have primarily been studied in the context of somatic pathogenic variants, with less focus on germline pathogenic variants. Therefore, it is crucial to comprehend the structural changes and molecular interactions of GIRK4 channels using protein modeling and simulation studies. In this work, we analyzed germline mutations in *KCNJ5* that lead to familial hyperaldosteronism type III (FH-III) in order to understand the mechanisms behind these pathogenic variants in affected patients. This work addresses the existing knowledge gap and enhances our understanding of this disorder, particularly at the molecular level. Unraveling the molecular mechanisms of *KCNJ5* mutations is essential for drug or molecule development that could stabilize the GIRK4 protein or modulate its activity.

This study builds on our previous GIRK4 modeling work but addresses a distinct mechanistic question. Previous studies focused on identifying candidate modulators of WT and GIRK4^G151E^ channels using docking-based screening approaches [16] and on examining the common G151R variant using similar screening strategies [17]. In contrast, the present study focuses on the germline GIRK4^G151E^ variant implicated in familial hyperaldosteronism type III. Here, we investigate how this substitution influences protein dynamics and ligand interactions by analyzing relative structural stability, residue-level flexibility, and small molecule-binding energetics.

## 2. Results

**Figure 1:**
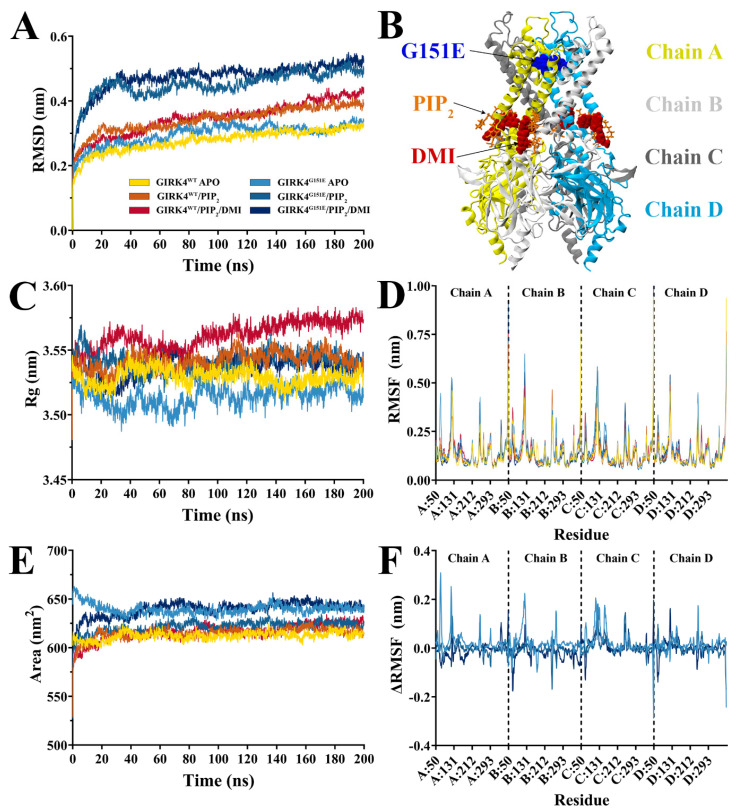
Molecular dynamics simulations of human GIRK4^WT^ and GIRK4^G151E^ in apo, PIP_2_-bound, and PIP_2_/DMI-bound states. Each system was simulated for 200 ns in triplicate, and values are shown as replicate-averaged trajectories. (**A**) Root mean square deviation (RMSD) of the protein backbone relative to the initial structure. Systems reached equilibrium after ~50 ns, after which G151E exhibited consistently higher RMSD values compared to WT across ligand-bound conditions, indicating reduced structural stability. Legend indicating different coloured lines corresponding to each system is applicable for entire figure. (**B**) Structural representation of the GIRK4^G151E^ homotetramer in complex with PIP_2_ and DMI, showing chain organization (**A**–**D**) and ligand placement. (**C**) Radius of gyration (Rg) of the protein backbone over time. Similar Rg values across systems indicate that overall protein compactness is maintained despite local structural differences. (**D**) Root mean square fluctuation (RMSF) of backbone atoms following equilibration, plotted by residue and separated by chain. Peaks correspond to regions of increased flexibility, with notable variability observed in extracellular and cytosolic domains. (**E**) Solvent-accessible surface area (SASA) over time. G151E systems show slightly increased SASA compared to WT, suggesting increased solvent exposure. Dashed lines indicate separate chains on the homotetramer. (**F**) Difference in RMSF (ΔRMSF), calculated as GIRK4^G151E^ − GIRK4^WT^ for each system. Positive values indicate increased flexibility in the mutant, highlighting regions potentially affected by the G151E substitution.

We performed classical MD simulations of the G protein-activated inward-rectifier potassium channel 4 (GIRK4) protein to examine its behavior in a dynamic environment (Figure 1). The wild-type [18] and G151E mutant GIRK4 proteins were studied as homotetramers in their unbound state (APO), coupled with the cofactor phosphatidylinositol-4,5-bisphosphate (PIP_2_), and bound with both the cofactor and a ligand 3-[2-(3,4-dimethoxyphenyl)-2-oxoethyl]-3-hydroxy-1-(1-naphthylmethyl)-1,3-dihydro-2H-indol-2-one (DMI) (PIP_2_/DMI) [19], as illustrated in Figure 1B. Simulations were performed in triplicate, and values are reported as mean ± SD.

A root mean square deviation (RMSD) analysis indicated that the systems were equilibrated after 50 ns (Figure 1A), so subsequent analysis was performed after this timepoint. The RMSD values were higher for G151E compared to WT when bound with the PIP_2_ and DMI, with average values of 0.46 ± 0.03 and 0.49 ± 0.02 nm for GIRK4^G151E^/PIP_2_ and GIRK4^G151E^/PIP_2_/DMI compared to 0.36 ± 0.02 and 0.37 ± 0.03 nm for GIRK4^WT^/PIP_2_ and GIRK4^WT^/PIP_2_/DMI, respectively. A statistical comparison using one-way ANOVA on replicate-averaged RMSD values indicated a significant difference between the systems (*p* < 0.05). This suggests that GIRK4^WT^ may be more stable than GIRK4^G151E^ systems. The average radii of gyration (Rg) were similar between the systems (3.53 ± 0.01 for GIRK4^WT^ APO; 3.51 ± 0.01 for GIRK4^G151E^ APO; 3.54 ± 0.01 for GIRK4^WT^, PIP_2_, GIRK4^G151E^ PIP_2_ and PIP_2_/DMI; and 3.56 ± 0.01 for GIRK4^WT^/PIP_2_/DMI), suggesting that the overall shape of the GIRK4 channel is maintained (Figure 1C). Solvent-accessible surface area (SASA) throughout the simulation was slightly higher for GIRK4^G151E^ systems (Figure 1E: 639.13 ± 3.89, 623.13 ± 3.71, and 623.45 ± 3.71 nm^2^ for GIRK4^G151E^ APO, PIP_2_, and PIP_2_/DMI compared to 613.10 ± 3.30, 616.50 ± 3.47, and 619.42 ± 5.39 nm^2^ for GIRK4^WT^ APO, PIP_2_, and PIP_2_/DMI systems, respectively). The larger SASA values for GIRK4^G151E^ compared to GIRK4^WT^ suggest that there is a larger surface area of the protein exposed to water, also indicative of the compromised stability of the mutant channel.

A root mean square fluctuation (RMSF) analysis showed that the largest fluctuations occurred in the extracellular region of GIRK4 at residues 120–125 in all the systems (Figure 1D), indicating that residues of this region are generally more flexible. Fluctuations were also observed in the pore-forming region around residues 144–146, with RMSF values of approximately 0.30 nm. GIRK4^WT^ values were subtracted from the corresponding GIRK4^G151E^ system and the differences were plotted as ΔRMSF (Figure 1F). Larger differences in RMSF between GIRK4^WT^ and GIRK4^G151E^ were observed in the extracellular domains around residues 118–126, which may have a role in the binding of peptide inhibitors such as tertiapin-Q [20]. There were also notable differences in the cytosolic domain at residues 68–71, located in proximity to PIP_2_ and DMI, with a ΔRMSF of 0.31 nm in chain A of APO and −0.16 nm in chain B of PIP_2_/DMI-bound GIRK4. A larger ΔRMSF of up to 0.17 nm was also observed in regions around residue 247, where E246K and G247R mutations in GIRK4 have been linked to primary aldosteronism [21].

**Figure 2:**
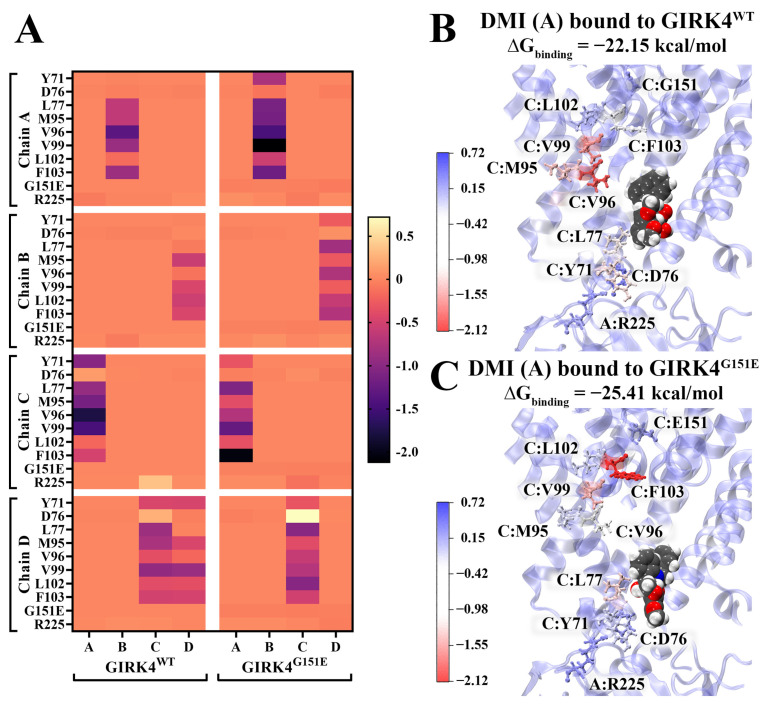
MM-PBSA analysis of 3-[2-(3,4-dimethoxyphenyl)-2-oxoethyl]-3-hydroxy-1-(1-naphthylmethyl)-1,3-dihydro-2H-indol-2-one (DMI) bound to G protein-activated inward-rectifier potassium channel 4 (GIRK4) protein. (**A**) Heatmap showing per-residue binding free energy contributions (kcal/mol) for DMI interaction with GIRK4^WT^ and GIRK4^G151E^ across chains A–D. Negative values (darker colors) indicate favorable contributions to binding, while positive values indicate unfavorable contributions. Consistent differences in residue-level contributions are observed between WT and G151E systems, particularly around residues involved in ligand interaction. (**B**,**C**) Representative binding poses of DMI in GIRK4^WT^ (**B**) and GIRK4^G151E^ (**C**), highlighting residues contributing to ligand binding. Residues are colored according to total energy contribution (kcal/mol), with labels indicating key interacting residues. DMI is shown in van der Waals representation, with carbon atoms displayed in gray, oxygen atoms in red, nitrogen atoms in blue, and hydrogen atoms in white. The G151E variant shows altered residue-level energetic contributions and a more favorable overall binding free energy (ΔG_binding = −25.41 kcal/mol) compared to WT (ΔG_binding = −22.15 kcal/mol), consistent with the MM-PBSA analysis. All energy contributions are shown in kcal/mol.

DMI was previously identified as a selective activator of human GIRK4 and thus was utilized to examine the effect of ligand binding [19]. DMI was selected as a structurally defined GIRK4-selective probe to test whether the G151E substitution changes the local binding environment and residue-level energetic contributions within a GIRK4-selective ligand-binding framework. MM-PBSA was used to calculate the binding free energy of DMI to the GIRK4^WT^ and GIRK4^G151E^ GIRK4 proteins. Overall, DMI binds more strongly to GIRK4^G151E^ than GIRK4^WT^, with van der Waals interactions being the predominant driving factor for binding (Table 1). Binding energy was decomposed on a per-residue basis for further analysis, depicted in Figure 2. The key residues are largely similar between GIRK4^WT^ and GIRK4^G151E^, with stronger contributions from F103 in GIRK4^G151E^ (Chain C: −0.5 kcal/mol for GIRK4^WT^; −2.05 kcal/mol for GIRK4^G151E^). L77, a major determinant of isoform-specific selectivity of drugs to GIRK4 [19], is shown here to have stronger binding energy contributions in GIRK4^G151E^ compared to GIRK4^WT^ (Figure 2).

**Figure 3:**
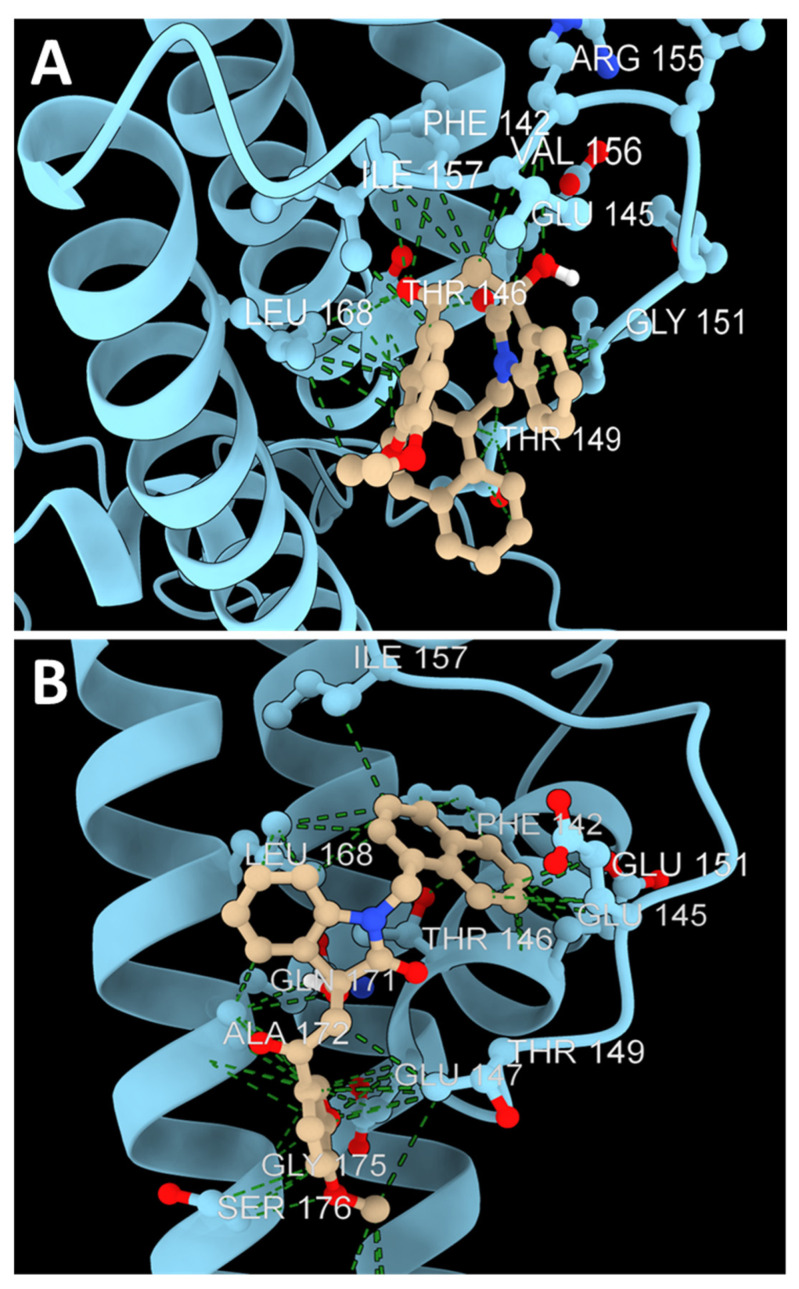
Molecular docking of DMI to (**A**) GIRK4^WT^ and (**B**) GIRK4^G151E^. DMI and GIRK4 residues in contact with DMI are shown in CPK representation, with GIRK4 shown in light blue and DMI shown in light brown. Oxygen atoms are red, hydrogen in white, and nitrogen in dark blue.

To identify the molecular-scale effects of selectivity-filter mutations, a secondary docking experiment with residue contact analysis was performed for GIRK4^WT^ and GIRK4^G151E^. This analysis provides another in silico experiment to confirm whether the MD results are consistent with data gathered from other software. This additional data is visualized in Figure 3.

The average affinity of the docking results in the correct site against wild-type GIRK4 was –5.726 kcal/mol. The average unbound RMSD of all the poses was 4.721 Å. The indole moiety appears to interact with the backbone atoms of Gly151 in the strongest docks; however, few other strong interactions exist in the best conformations of this type, resulting in lower affinity in comparison to the GIRK4 mutants. However, all the poses of this dock are consistent with one another, which could indicate selectivity for this site over others.

The average affinity of the docks in the correct site for GIRK4^G151E^ was –6.749 kcal/mol. The average unbound RMSD of all the poses was 13.11 Å. In the strongest pose with GIRK4^G151E^, the naphthalene group interacts with the substituted glutamic acid and is further stabilized in this position through contacts with three neighboring nonpolar residues: Isoleucine 157, Leucine 168, and Phenylalanine 142. This dock shows the highest average affinity of any conducted in this study, suggesting a unique reactive domain that will function much differently than its wild-type counterpart.

## 3. Discussion

FH-III is a distinct type of primary aldosteronism characterized by its clinical presentation and biochemical nature. Medical therapies are currently nonspecific, and clinicians still face challenges in controlling blood pressure well in these patients.

**Figure 4:**
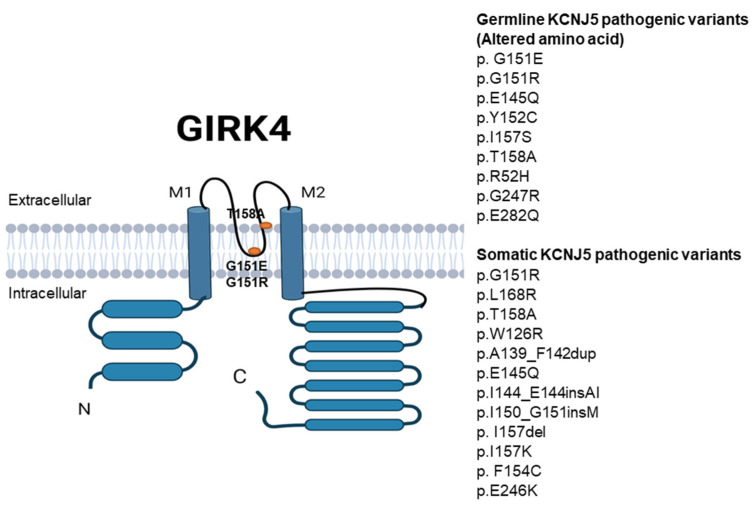
GIRK4 channel structure and germline and somatic mutations previously reported. A schematic representation showing the structure of the mammalian GIRK4 (Kir3.4) channel. The channel is composed of two domains: the transmembrane domain with two helices (M1 and M2) and selectivity filter, and the cytoplasmic domain with cytoplasmic pore, N- and C-termini with corresponding sequences important for GIRK4 signaling. *KCNJ5* mutations (e.g., G151E, G151 R, and T158A) are located within or near the selectivity filter. These mutations result in the loss of potassium Kir3.4 channel selectivity and sodium entry, resulting in cell depolarization, intracellular calcium entry, and excess aldosterone synthesis. Germline and somatic mutations previously reported are listed.

Our work highlights the mechanisms of GIRK4 channel pathogenic variants in FH-III and explores the GIRK channel defect and its potential relevance to treating hypertension using protein modeling. The *KCNJ5* gene is particularly important because its mutations are the most common cause of PA. *KCNJ5* encodes a membrane protein of 419 amino acids, GIRK4. GIRK4 is an inwardly rectifying potassium channel in vivo that forms homotetramers and heterotetrameric complexes based on protein biochemistry and crystal structure analysis. The structure of GIRK4 is shown in Figure 4. *KCNJ5* pathogenic variants are present in germline or somatic mutations (Figure 4). While the mechanism of mutations was studied in *KCNJ5* somatic mutations, little data is available on germline *KCNJ5* mutations.

Understanding the physiology of regulation of GIRK4 channels is crucial for modeling and drug discovery for this disease. The regulation of GIRK4 channels is complex and can vary according to the cell type. GIRK4 channels can be activated or inhibited by different factors or compounds [15]. In this simulation, we used phosphatidylinositol 4,5-bisphosphate (PIP2) as a cofactor that is necessary for GIRK4 activation [22]. Phosphorylation of GIRK4 channels (e.g., protein kinases) can either increase or decrease their open probability depending on the specific kinase and the phosphorylation site. Other various allosteric GIRK channel activators include intracellular Na^+^ and low-molecular-weight alcohols (e.g., methanol and ethanol) [23], where an increase in intracellular Na^+^ concentration leads to the activation of GIRK4-containing channels, strengthening PIP_2_ binding or increasing receptor affinity for Gβγ. In this study, WT and G151E systems had higher average RMSD values when bound with PIP2 compared to the apo state (Figure 1A), indicative of structural rearrangements that may correspond to channel activation. However, further simulations spanning a longer timescale would be required to assess the impact of these structural changes on Gβγ affinity.

*KCNJ5* G151E affects the selectivity pore of GIRK4. Mutations affecting GIRK4 localize near or within the selectivity filter, such as p.Gly151Glu, p.Gly151Arg, p.Thr158Ala, and p.Leu168Arg, which induce a change in the ion selectivity of the channel (Figure 4). The mutations localized far away from the selectivity filter (p.Arg115Trp and p.Glu246Gly) do not change the channel selectivity but decrease the abundance of the mutated channels at the membrane. Interestingly, patients with the *KCNJ5* G151E mutation have hypertension with normal adrenal gland structure (no adrenal masses). In contrast, patients with germline *KCNJ5* G151R mutations present with a severe form of PA and extensive adrenocortical hyperplasia requiring bilateral adrenalectomy. The increased sodium ion conductance of the G151E mutated channel found greater cell death induced by G151E relative to G151R. The *KCNJ5* mutations cause sodium ion conductance due to the loss of selectivity for potassium ions by the channel pore. In adrenocortical cells, the consequent membrane depolarization triggers the opening of voltage-gated calcium channels, and calcium ion influx ultimately activates aldosterone production. The selectivity-filter mutations presented replace a nonpolar glycine with very reactive charged amino acids that drastically alter the electrostatic landscape of the domain. The consequence of these mutations is a depolarization of the membrane through an uncontrolled flux of sodium ions [11], which, in turn, triggers the synthesis of aldosterone as a response [15]. Understanding the atomic-scale mechanisms of these pathogenic mutations and how they respond differently to newly discovered ligands is an important first step in more particular and targeted medicinal chemistry studies.

Here, an important mechanism of GIRK4 dysfunction is shown in silico. Molecular dynamics simulation is a method to assess the movement of atoms and molecules over a period of time. Residue fluctuation refers to the variability of amino acid residue movement within its structure. Understanding how residues move or fluctuate provides crucial insight into protein dynamics and behavior. Protein fluctuation is determined by interactions with surrounding molecules and thermal motions. Residue fluctuation plays a key role in ligand binding and protein–protein interactions. Crystallography and NMR are used to assess protein dynamics. The WT and G151E mutant GIRK4 proteins were studied as homotetramers in their unbound and bound states, as highlighted in the results. The RMSD of G151E systems had higher values compared to WT, where smaller deviations indicate a more stable protein structure. Similarly, G151E systems had higher SASA than WT, highlighting that the stability of the protein structure is compromised when pathogenic mutations occur.

Overall, it was observed that structural rearrangements occur in GIRK4^G151E^ when compared to GIRK4^WT^, displaying higher RMSD and SASA, which may be partially attributed to differences in the residue fluctuations in the cytosolic and extracellular domains. A larger ΔRMSF was observed in the regions surrounding residue 247, where E246K (somatic) and G247R (germline) mutations in GIRK4 have been linked to primary aldosteronism [21] (Figure 4). ΔRMSF is interpreted here as a measure of altered residue-level flexibility rather than a direct indicator of functional change. Accordingly, the observed differences do not establish functional consequences but instead highlight regions that may be more dynamically perturbed in the G151E variant. These findings therefore prioritize specific residues and domains for future experimental validation.

The MM-PBSA analysis suggests that ligands may demonstrate a stronger affinity for the mutant GIRK4^G151E^ compared to WT. DMI is a highly selective modulator of GIRK4, with residue L77 playing a role in determining isoform-specific selectivity [19]. In the binding of DMI, residue L77 exhibits a stronger energy contribution in GIRK4^G151E^ compared to GIRK4^WT^. These findings highlight variant-specific differences in ligand-binding interactions and may inform future studies of GIRK4-targeted modulation. It should be noted that DMI is used here as a structural probe of ligand binding, and the present results do not imply therapeutic suitability.

The trajectories used here are appropriate for comparing early local relaxation, relative stability, residue-level flexibility, SASA, and ligand-contact patterns under matched conditions, but they are not sufficient to define rare gating events or the full free-energy landscape of GIRK4 channel opening. Accordingly, we interpret the MD data as evidence of altered local conformational dynamics around the G151E substitution and associated domains rather than as direct proof of a complete gating mechanism. Future work should integrate enhanced-sampling MD with experimental validation in adrenal zona glomerulosa cell models.

The principal contribution of this study is a matched replicate MD comparison of GIRK4^WT^ and the germline GIRK4^G151E^ variant in apo, PIP2-bound, and PIP2/DMI-bound states. The results suggest that G151E increases local structural instability and solvent exposure while altering residue-level ligand-contact energetics. These findings extend prior docking-focused studies by providing a dynamic framework for identifying and prioritizing residues and ligand-binding regions for further investigation.

Deciphering the molecular mechanism of *KCNJ5* mutations is crucial for drug discovery to treat patients with FH type III. These small molecules or drugs can interact with and stabilize the affected residues in the GIRK4 protein to modulate its activities.

In conclusion, we simulated mutations and modulators of GIRK channels, which can serve as a promising preclinical model. Our clinical experience and GIRK4 modeling in FH-III form the foundation for understanding the channel structure in mutations, which can be the first steps in developing a pharmacological toolkit and promising therapies.

## 4. Methods

### 4.1. Homology Modeling

**Figure 5:**
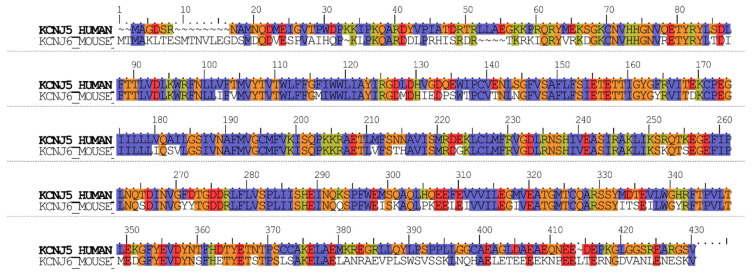
Sequence alignment of human GIRK4, encoded by *KCNJ5* (UniProt ID: P48544), and mouse GIRK2, encoded by *KCNJ6* (UniProt ID: P48542). Matching residues are colored according to residue type: hydrophobic in blue, acidic in red, basic in green, and other in orange. Sequence alignment was performed using ClustalW in Maestro 12.3.013 with default parameters [27].

Homology modeling was used to construct the protein for simulations, as recently described [16]. As there was no structure of human GIRK4 available, a homology model was constructed in this study. The amino acid sequence of human *KCNJ5* encoding GIRK4 was retrieved from the UniProt database (ID: P48544) [24]. Template identification was performed using NCBI Protein BLAST+ 2.17.0 against the Protein Data Bank (PDB) [25], and the template was selected based on sequence identity, alignment coverage, and structural resolution. The cryo-EM structure of mouse GIRK2 (PDB ID: 6XIT) was used as the template for the homology model, encompassing residues 50–573, with a resolution of 3.3 Å and sequence identity of 83.13% [26]. Alignment of the full sequences was performed using ClustalW in Maestro 12.3.013 with default parameters and is shown in Figure 5 [27].

The homology model was generated as a homotetramer using the SWISS-MODEL server [28], and stereochemical quality was evaluated using PROCHECK SAVES v6.1 [29]. The Ramachandran plot showed 91.5% of residues located in the most favored regions, 8.1% of residues located in the additional regions, and 0.3% of residues located in the generously allowed regions [16]. The model preserved the canonical Kir selectivity-filter region and the PIP2-binding/basic-residue architecture, whereas the most variable regions relative to the template occurred in extracellular loops and portions of the cytosolic domain. To generate the G151E variant, the mutation was introduced on all four chains of wild-type [18] GIRK4 using the mutagenesis tool in PyMOL v1.7.4.5 [30].

### 4.2. Molecular Docking

The coordinates for phosphatidylinositol-4,5-bisphosphate (PIP_2_) molecules were obtained by modifying the co-crystallized cofactors from the template [16]. Starting coordinates for 3-[2-(3,4-dimethoxyphenyl)-2-oxoethyl]-3-hydroxy-1-(1-naphthylmethyl)-1,3-dihydro-2H-indol-2-one (DMI) were obtained through molecular docking. The structure was downloaded from the National Center for Biotechnology Information PubChem Database (CID: 2915602) [31]. Protein and ligand structures were prepared using AutoDockTools v1.5.6 [32]. WT and G151E GIRK4 structures were prepared as macromolecules, and DMI as a ligand with all rotatable bonds was activated. The binding site was defined around active residues in proximity to the PIP_2_ head group for each chain [19]. For docking, a 30 × 30 × 30 Å grid box was centered around residues K59, K85, K194, and K195 in each chain. These residues were selected based on their proximity to the PIP2 head group and previously reported ligand interaction regions, providing a biologically relevant binding environment for docking. Docking simulations were performed using AutoDock Vina v1.1.2 [33] at an exhaustiveness of 2048 to generate a maximum of 20 binding modes per ligand. The grid dimensions and high exhaustiveness parameter were chosen to ensure comprehensive sampling of the binding site and to reduce stochastic variation in docking results. The top binding mode aligning with conformations similar to those proposed by Cui et al., i.e., forming hydrogen bonds with W86 and K195 [19], was selected as the starting structure for subsequent molecular dynamics simulations. Ligand topology and parameter files were generated using the CHARMM General Force Field (CGenFF) Program v4.6 [34].

A secondary molecular docking was performed to confirm the results using different software. This independent docking approach was included to assess the robustness of the predicted binding mode and to minimize software-specific bias. ColabFold [35] was used to dock DMI to GIRK4 and its mutant in a site-specific manner. A 36 × 25 × 27 Å docking grid was centered around residue 151 for all proteins and run with an exhaustiveness of 8. The average RMSD of the top poses for each dock was calculated along with the average binding affinity of the poses in the correct site. The contacted residues and hydrogen bonds were found and visualized using UCSF ChimeraX [36].

### 4.3. Molecular Dynamics (MD) Simulations

MD simulations were performed using GROMACS 2018.2 [37,38], as previously described [39]. The CHARMM36 force field was utilized [40] with the TIP3P water model [41]. A total of six systems were examined: homotetrameric GIRK4^WT^ and GIRK4^G151E^ complexes as the ligand-free protein (APO), bound with the cofactor PIP_2_, and in complex with both PIP_2_ and the small molecule DMI (PIP_2_/DMI). Systems were solvated in a triclinic box under periodic boundary conditions with a minimum distance of 1.0 nm between any protein atom and the box edge, then neutralized and salted with 0.15 M NaCl. Energy minimization was performed using the steepest-descent gradient method. Canonical (NVT) equilibration was performed for 100 ps at a temperature of 310 K with a modified Berendsen thermostat [42], followed by 100 ps under an isothermal–isobaric (NPT) ensemble for 100 ps with the Parrinello–Rahman barostat [43] maintaining pressure at 1.0 bar. Bond lengths were constrained using the LINCS algorithm [44], and long-range electrostatic forces were calculated using particle-mesh Ewald (PME) scheme [45] (grid spacing 0.16 nm). Cut-off ratios of 1.2 nm were used for Coulomb and van der Waals potentials. Production runs were carried out with a time-step of 2 fs for 200 ns in triplicate. Independent replicate simulations were initiated with different random velocity seeds to ensure statistical independence between trajectories. Statistical analysis was performed using GraphPad Prism version 11.0.0 for Windows (GraphPad Software, Boston, MA, USA).

### 4.4. Trajectory Analysis and MM-PBSA

Analysis of the trajectories was performed using gmx rms, gms rmsf, gmx gyrate, and gmx sasa within GROMACS. The molecular mechanics Poisson–Boltzmann surface area (MM-PBSA) method was used to calculate the binding free energy of DMI to GIRK4 using g_mmpbsa v5.1.2 [46]. MM-PBSA calculations were performed on the final 20 ns of the trajectories at 100 ps intervals. The final 20 ns of each trajectory was selected for analysis to ensure sampling from equilibrated regions of the simulation. Energy contributions from electrostatic, van der Waals, and polar solvation terms were calculated using the adaptive Poisson–Boltzmann Solver (APBS) v1.3 [47]. Grid spacing was set to 0.05 nm, and values of 80 and 2 were used for the solvent and solute dielectric constants, respectively. The dielectric constants for the solvent (80) and solute (2) were selected based on standard values commonly used in MM-PBSA calculations to approximate aqueous and protein environments, respectively. Solvent-accessible surface area (SASA) was used to approximate the nonpolar energy contribution, with the probe radius set to 0.14 nm. Entropic energy terms were excluded from the calculations. Complexes were visualized using VMD 1.9.3 [22].

## Figures and Tables

**Table 1:** Summary of binding free energy contribution terms *.

Energy Terms	GIRK4^WT^				GIRK4^G151E^			
(kcal/mol)	A	B	C	D	A	B	C	D
ΔE_vdW_	−30.80 ± 7.66	−23.00 ± 3.62	−33.36 ± 1.20	−28.03 ± 2.70	−31.33 ± 1.67	−32.03 ± 7.77	−28.65 ± 0.67	−29.99 ± 5.03
ΔE_elec_	−2.02 ± 0.68	−1.23 ± 0.28	−4.76 ± 2.64	−1.10 ± 0.53	−3.86 ± 2.27	−2.08 ± 1.11	−4.65 ± 2.50	−3.96 ± 1.90
ΔG_polar_	14.55 ± 6.44	9.71 ± 1.23	18.91 ± 3.05	12.52 ± 2.23	13.61 ± 1.16	13.42 ± 4.93	14.07 ± 1.97	13.42 ± 3.67
ΔG_nonpolar_	−3.88 ± 0.79	−3.06 ± 0.40	−4.19 ± 0.20	−3.61 ± 0.44	−3.84 ± 0.35	−3.93 ± 0.86	−3.49 ± 0.14	−3.88 ± 0.79
ΔG_binding_	−22.15 ± 3.29	−17.59 ± 2.75	−23.41 ± 1.98	−20.19 ± 1.17	−25.41 ± 3.04	−24.60 ± 5.50	−22.73 ± 1.07	−24.42 ± 3.62

* Energies are shown in kcal/mol along with standard deviation for the binding of four molecules of 3-[2-(3,4-dimethoxyphenyl)-2-oxoethyl]-3-hydroxy-1-(1-naphthylmethyl)-1,3-dihydro-2H-indol-2-one (DMI) to human G protein-activated inward-rectifier potassium channel 4 (GIRK4). ΔE_vdW_ is the van der Waals interaction, ΔE_elec_ is the electrostatic interaction, ΔG_polar_ is the polar contribution, ΔG_nonpolar_ is the nonpolar contribution to the solvation free energy estimated by solvent-accessible surface area (SASA), and ΔG_binding_ is the binding free energy.

## Data Availability

The data presented in this study are available on request from the corresponding author.

## Abbreviations

FH-III	Familial hyperaldosteronism type III
GIRK	G protein-activated inward-rectifier potassium channel
MD	Molecular dynamics
PA	Primary aldosteronism
PIP2	Phosphatidylinositol-4,5-bisphosphate
RMSD	Root mean square deviation
